# Multilineage differentiation of human bone marrow mesenchymal stem cells *in vitro* and *in vivo*

**DOI:** 10.3892/etm.2013.1042

**Published:** 2013-04-02

**Authors:** YOU-HUA ZHENG, WEI XIONG, KAI SU, SHI-JUN KUANG, ZHI-GUANG ZHANG

**Affiliations:** Department of Oral and Maxillofacial Surgery, Guanghua School of Stomatology, Hospital of Stomatology, Sun Yat-Sen University, Guangzhou, Guangdong 510055, P.R. China

**Keywords:** bone marrow-derived mesenchymal stem cells, differentiation, osteoblast, tissue engineering

## Abstract

The aim of the present study was to investigate the ability of human bone marrow-derived mesenchymal stem cells (BMSCs) to undergo multilineage differentiation. Human BMSCs were isolated from the ilia of donors by density gradient centrifugation, then purified by adherent separation and cultured *in vitro*. P3 or P4 BMSC populations were collected and induced for multilineage differentiation into osteoblasts, adipocytes and neuroblasts using an inductive medium *in vitro*. The BMSCs were cultured in either an osteoblast or chondroblast induction medium, seeded onto porous coral scaffolds and implanted into mice *in vivo*. The mice were sacrificed by anesthesia overdose at 6 or 9 weeks post-surgery. The scaffolds were then removed for analysis. Lipid vacuoles were observed subsequent to being cultured in an adipogenic medium. These accumulated lipid vacuoles were detected using Sudan Black B and Oil Red O (positive) staining. Deposited calcium was detected using von Kossa and Alizarin Red S (positive) staining subsequent to being cultured in an osteogenic medium. The BMSCs retracted to form neuron-like cells with axon- and dendrite-like processes following induction by β-mercaptoethanol. The cells were positively stained by toluidine blue and glial fibrillary acidic protein (GFAP) immunohistochemistry. Newly formed bone tissues were observed and islands of cartilage tissue were also formed at 9 weeks post-implantation *in vivo*. The present study demonstrated that human BMSCs were homogeneous and differentiated with high fidelity to osteogenic, adipogenic, neurogenic or chondrogenic lineages. These cells also form bone and cartilage tissues when implanted *in vivo* and may therefore be used as seed cells in bone tissue engineering.

## Introduction

Adult stem cells, although not totipotent, are able to differentiate into specific precursor and terminal cells. Bone marrow-derived mesenchymal stem cells (BMSCs), as seed cells for bone tissue engineering, possess the generality of stem cells with self-replicating, amplification and multilineage differentiation potential. *In vitro* and *in vivo* studies have indicated that culture-expanded BMSCs are capable of differentiation along osteogenic, chondrogenic and adipogenic lineages, as well as into cardiomyocytes, skeletal muscle and neural precursors (1–9). In the present study, BMSCs were induced to differentiate into osteogenic, adipogenic and neural lineages *in vitro*. The cells were then implanted into subcutaneous pockets on the dorsa of nude mice to form new bone and cartilage tissues.

## Materials and methods

### Isolation and culture of human BMSCs

The BMSCs were isolated using our previously described methods (10). Briefly, bone marrow (10 ml) was obtained from the iliac crest of voluntary donors from whom informed consent had been obtained. The aspirate was diluted at a ratio of 1:2 in Dulbecco’s modified Eagle’s medium-low glucose (DMEM-LG; Gibco, Carlsbad, CA, USA). The mononuclear cell layer was removed from the interface, washed twice and suspended in DMEM at 10^7^ cells/ml subsequent to density gradient centrifugation (density of 1.077 g/ml, Ficoll) at 400 × g for 20 min. Each 25-cm^2^ flask (Corning Inc., One Riverfront Plaza, Corning, NY, USA) contained DMEM with 10% fetal bovine serum (FBS; Gibco) supplemented with 1% penicillin/streptomycin (Gibco). The non-adherent cells were discarded and the adherent cells were washed with phosphate-buffered saline (PBS; Gibco) on the second day. The cells were then cultured in DMEM with antibiotics and 10% FBS in a humidified incubator (37°C, 5% CO_2_) with renewal of the culture medium every 3 days. The medium containing 10% FBS was replaced every 3 or 4 days. At ∼50% confluence, the cells were suspended using a 0.25% trypsin/0.02% EDTA solution (Sigma, St. Louis, MO, USA) and replated at ∼5,000 cells/cm^2^. The cells were split every 5–7 days following the first passage. The cells were subsequently subjected to analysis following three or four passages.

### Multilineage differentiation assays in vitro

Osteogenic, adipogenic, neurogenic and chondrogenic differentiation were induced according to the reported methods (6), with certain modifications.

### Osteogenic differentiation

Three or four passages of BMSCs were adjusted to a concentration of 1×10^5^/ml and cultured on a 6-well culture plate (1 ml/well). The cells were cultured in a humidified incubator (37°C, 5% CO_2_) with renewal of the culture medium every 3 days. The cells were incubated in a differentiation medium for 2–4 weeks once the cells had reached 100% confluence, during which time the medium was changed every 2–3 days. The differentiation medium was as follows: DMEM-LG supplemented with 10% FBS, 1 *μ*M dexamethasone (Sigma), 50 *μ*g/ml ascorbic acid (Sigma), 10 mM sodium β-glycerophosphate (Sigma) and 1% penicillin/streptomycin (Sigma). The cells were fixed with ice-cold 70% ethanol and stained with Alizarin Red S (Amresco, Solon, OH, USA), as well as the von Kossa stain, to detect mineralization (calcium deposits). The alkaline phosphatase (ALP) activity was also tested.

### Adipogenic differentiation

The cells were first grown to 100% confluence and then incubated for 3 days in an induction medium consisting of DMEM-LG supplemented with 10% FBS, 100 *μ*M indomethacin (Sigma), 0.1 *μ*M dexamethasone, 0.5 mM 3-isobutyl-1-methylxanthine (IBMX, Sigma), 10 *μ*g/ml human insulin (Sigma) and 1% penicillin/streptomycin. The cells were incubated in the induction and maintenance media for >2 weeks and then fixed with 4% paraformaldehyde for 30 min at room temperature and stained with Oil Red O, as well as Sudan Black B (Amresco), to detect fat deposition.

### Neurogenic differentiation

The cells were grown to 100% confluence and then incubated for 24 h in a pre-induction medium consisting of DMEM-LG supplemented with 20% FBS and 1 mM/l β-mercaptoethanol (BME), followed by incubation for 5 h in an induction medium consisting of DMEM-LG supplemented with 5 mM/l BME. The neuroblasts were examined by toluidine blue staining and glial fibrillary acidic protein (GFAP) immunohistochemical staining.

### Chondrogenic differentiation

Three or four passages of BMSCs were adjusted to a concentration of 1×10^6^/ml and cultured in a 75-cm^2^ flask using a humidified incubator (37°C, 5% CO_2_) with renewal of the culture medium every 3 days. The cells were incubated in a differentiation medium for 10 days once the cells had reached 100% confluence, during which time the medium was changed every 2–3 days. The differentiation medium comprised DMEM-LG supplemented with 10% FBS, 10 ng/ml transforming growth factor-β1 (TGF-β1; Sigma), 6.25 *μ*g/ml insulin, 6.25 *μ*g/ml transferrin (Sigma), 0.1 *μ*M dexamethasone, 50 *μ*g/ml ascorbic acid and 1% penicillin/streptomycin.

### Osteogenesis and cartilage tissue formation in vivo

Three or four passages of BMSCs were incubated in an osteogenic and a chondrogenic differentiation medium for 10 days, respectively, during which time the medium was changed every 2–3 days. A 300-*μ*l cell suspension (4×10^7^ cells/ml, osteoblast or chondroblast) was used to inoculate multiple sites of a coral scaffold which was placed in an incubator for 2 days prior to implantation. The composites of the osteoblast or chondroblast coral scaffolds were then implanted into subcutaneous pockets on the dorsa of nude mice to form new bone and cartilage tissues. All *in vivo* mouse implantation experiments were performed in accordance with our institutional guidelines for animal care and use. A total of 18 nude mice (Guangzhou University of Chinese Medicine, Guangzhou, China) were randomly divided into three groups (n=6 animals/group), namely the osteoblast- and chondroblast-scaffold groups and the cell-free scaffold group. The mice were sacrificed by anesthesia overdose at 6 or 9 weeks post-surgery. The scaffolds were then removed for analysis. The implanted scaffolds were assessed using radiographic, histological and immunohistochemical methods.

## Results

### Osteogenic differentiation

The BMSCs were isolated by density gradient centrifugation and purified by adherent separation to obtain an ample amount of the BMSCs with a uniform appearance.

**Figure 1:**
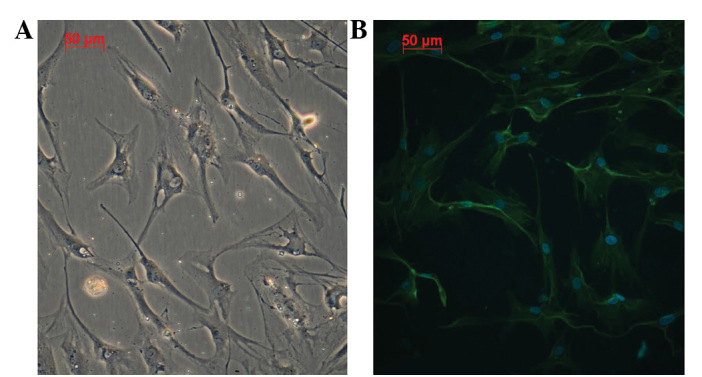
Following incubation in osteogenic medium for 5 days, the (A) appearance and (B) cytoskeleton of the BMSCs changed (magnification, ×200). BMSCs, bone marrow-derived mesenchymal stem cells.

**Figure 2:**
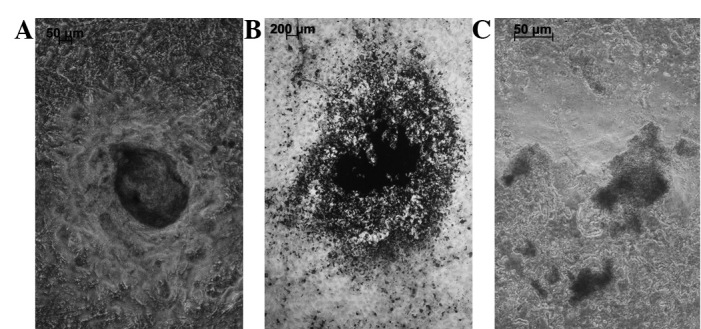
BMSCs induced into osteoblasts. Observed on (A) day 14 and (B) day 21, by von Kossa staining (magnification, ×200 and ×50, respectively); and (C) on day 14, by Alizarin Red staining (magnification, ×200). BMSCs, bone marrow-derived mesenchymal stem cells.

The cells were cultured in a differentiation medium for 2–4 weeks to induce osteogenic differentiation. The appearance of the cells changed in the first 3–5 days from long spindles to polygonal or irregularly shaped conformations (Fig. 1A). A cluster of cells demonstrating a growth tendency was present and a visible change to the cytoskeleton was observed (Fig. 1B). The deposition of calcium, an indicator of osteogenic differentiation, was determined by von Kossa and Alizarin Red S staining following 2–3 weeks of incubation in a differentiation medium. The von Kossa staining showed an uneven, black-stained calcified nodule with an unclear boundary (Fig. 2A and B). Alizarin Red S staining occurred in the sedimentary sections, indicating the deposition of calcium (Fig. 2C). An assay for ALP activity, an independent indicator of osteoblast differentiation, was conducted following the induction of the differentiation process. The results showed that the mean ALP activity increased moderately between days 1 and 7 and further increased between days 7 and 14, reaching a peak on day 14 and declining thereafter. The results of optical density were as follows (mean ± SD): day 1, 0.082±0.004; day 7, 0.171±0.008; day 14, 0.467±0.014; and day 28, 0.301±0.037. These results strongly suggest that BMSCs are able to differentiate into osteogenic cells.

### Adipogenic differentiation

**Figure 3:**
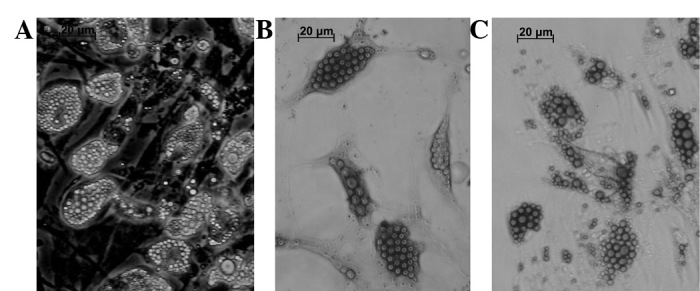
BMSCs induced into adipocytes. (A) Unstained, (B) stained with Sudan Black B; and (C) stained with Oil Red O; magnification, ×400. BMSCs, bone marrow-derived mesenchymal stem cells.

The cells were incubated in an induction medium for 2 weeks to induce adipogenic differentiation. The cells were stained with Oil Red O and Sudan Black B to detect lipid production. The cells changed in appearance from long spindles to polygonal shapes and became enlarged following 3–5 days incubation in a differentiation medium (Fig. 3A). Certain cells became round and spherical on day 7 and round, translucent lipid drops were observed in the cytoplasm. Numerous cells containing abundant lipids (adipocytes, Fig. 3A) were observed following 14 days of adipogenic differentiation. Positive staining with Sudan Black B and Oil Red O was observed (Fig. 3B and C). Lipid drops were distributed inside and outside of the cytoplasm as revealed by the Sudan Black B (Fig. 3B) and Oil Red (Fig. 3C) staining. In total, >50% of the adipocytes were induced in the cell populations.

### Neurogenic differentiation

**Figure 4:**
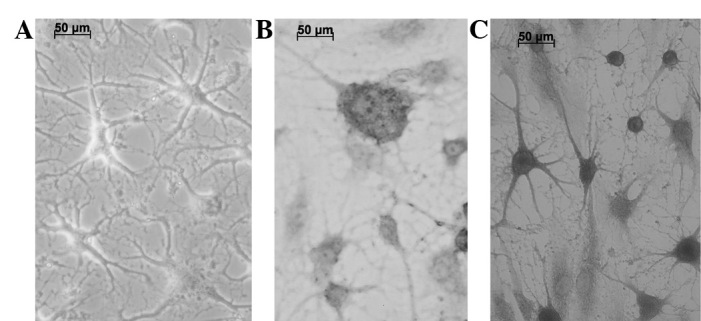
BMSCs induced into neuron-like cells. (A) Unstained, (B) stained with toluidine blue and (C) GFAP immunohistochemistry, magnification, ×200. BMSCs, bone marrow-derived mesenchymal stem cells; GFAP, glial fibrillary acidic protein.

The BMSCs were incubated in a pre-induction medium for 24 h and then in an induction medium for 5 h to induce neurogenic differentiation. The cells withdrew to form neuron-like cells with axon- and dendrite-like processes, instead of the spindle shapes (Fig. 4A). These cells presented a strong refractive trait, the Nissl bodies were displayed as a deep blue by toluidine blue staining (Fig. 4B) and the nucleus was nearly colorless. Positive immunohistochemical staining for GFAP was observed (Fig. 4C).

### Osteogenesis and cartilage tissue formation in vivo

**Figure 5:**
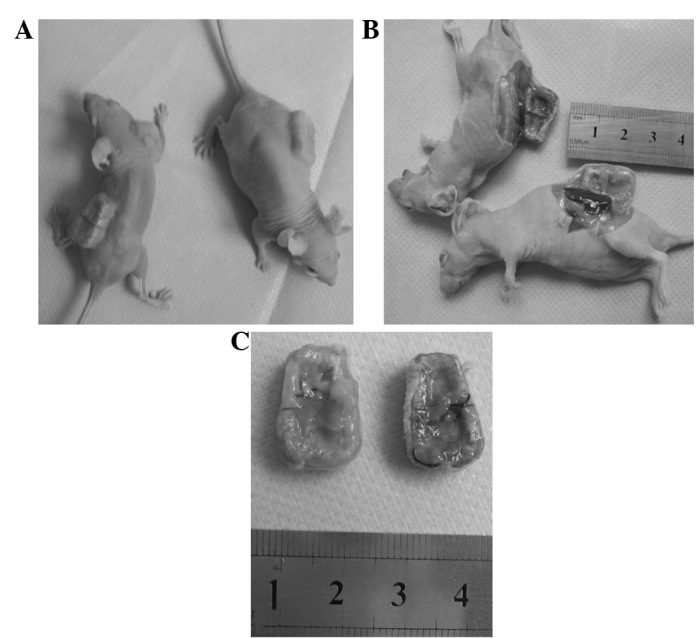
Findings of the visual inspection. The *in vivo* scaffold specimens maintained the initial shape of the coral scaffold. (A) Scaffold specimens on the dorsum of nude; left, osteoblast-scaffold, dark red, hard and bone-like; right, chondroblast-scaffold, translucent surface resembled cartilage. (B) Scaffold specimens of sacrificed mice; left, osteoblast-scaffold; right, chondroblast-scaffold. (C) removed scaffolds; left, chondroblast-scaffold; right, osteoblast-scaffold.

**Figure 6:**
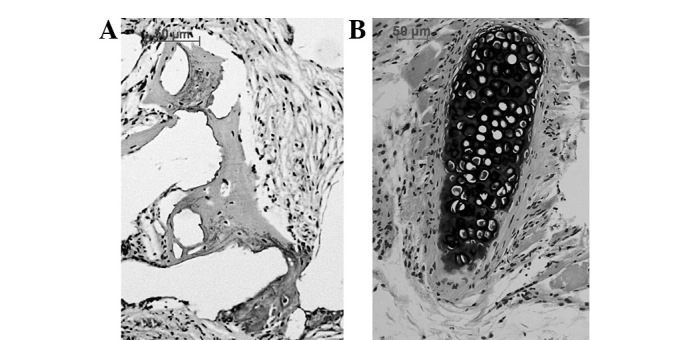
Microscopic evaluation demonstrated that (A) new trabecular bone and (B) hyaline cartilage formed 9 weeks post-implantation (H&E staining; magnification, ×200).

The visual inspection and X-ray results showed that the *in vivo* scaffold specimens in all three groups maintained the initial shape of the coral scaffold. The scaffold specimen was dark red, hard and bonelike in the osteoblast-scaffold group (Fig. 5). By contrast, the scaffold specimen had a translucent surface, resembling cartilage, in the chondroblast-scaffold group (Fig. 5). However, in the cell-free scaffold group, the scaffold specimen displayed only fibrous coral tissue growth. H&E staining indicated new bone formation but no new cartilage was formed in the osteoblast-scaffold group (Fig. 6A). Islands of cartilage tissue (Fig. 6B) were present in the chondroblast-scaffold group. The distribution and arrangement of the new bone and island cartilage tissues was disordered. The cell-free scaffold group displayed only host cell growth within the pores of the scaffold but no bone or cartilage tissues were observed. The immunohistochemical stain demonstrated that the newly formed bone displayed type I collagen expression in contrast to the type II collagen expressed by the cartilage.

## Discussion

Osteogenic, chondrogenic and adipogenic differentiation have been the most common methods used to identify whether analyzed cell populations are capable of multilineage differentiation. Pittenger *et al*(6) utilized clonally derived human mesenchymal stem cells (hMSCs) and osteogenic, adipogenic and chondrogenic differentiation assays to demonstrate that clonally derived hMSCs undergo differentiation to these three lineages. Of the six tested clonally derived populations, three (50%) differentiated into all three lineages, whereas two populations differentiated into the adipogenic and osteogenic lineages and one population became only osteogenic. These results demonstrated that BMSCs are capable of multilineage differentiation. Sudo *et al*(8) identified that the majority of the distinct populations of primary fibroblast-like cells (MPCs or MSCs) derived from various human tissues, including the lung, skin, umbilical cord and amniotic membrane tissues, contained cells that are able to differentiate into at least one mesenchymal lineage, including osteoblasts, chondrocytes and adipocytes.

A previous study has indicated that the expression of genes and proteins related to osteoblasts, including ALP, bone morphogenic proteins, osteocalcin, bone connexins and osteopontin receptors, are detectable in BMSCs incubated in a differentiated medium for 2–4 weeks. Newly formed bone was identified 6 weeks subsequent to the BMSCs being seeded to a biomaterial and implanted into nude mice. The ability of the various cell populations to differentiate into particular lineages appears to depend on the source tissue and induction conditions (8). Chemical inducers, including dexamethasone, β-glycerophosphate (β-GP) and ascorbic acid, are essential to cause MSCs to differentiate into osteoblasts (11–14). Dexamethasone may promote MSC differentiation, as well as osteocalcin and osteopontin expression, by raising the cAMP level of MSCs in response to parathyroid hormone and prostaglandin E2. This chemical inducer is also able to stimulate osteoblast-like cells to increase insulin-like growth factor secretion, collagen synthesis and ALP activity (15). β-GP, as an ALP substrate, provides phosphate ions, activates ALP activity, promotes the transformation of inorganic phosphorus to organophosphate and accelerates the formation of a mineralized extracellular matrix, as well as mineral deposits.

Several studies have compared the osteogenic and chondrogenic differentiation capacity of BMSCs to those of adipose-derived stem cells (ADSCs), as well as MSCs derived from peripheral blood and umbilical cord matrices (7,16–25), with varying results. The majority of the previous research findings showed that BMSCs were more advantageous than ADSCs (7,17,18,21–24). Afizah *et al*(16) compared the chondrogenic potential of human BMSCs with that of ADSCs from the same donors. Qualitative and quantitative methods were used to assess for variations in the expression of cartilage markers at the gene and protein levels. The findings suggested that BMSCs were more suitable than ADSCs for chondrogenesis. Huang *et al*(21) compared the chondrogenic potential of progenitor cells isolated from bone marrow aspirates and adipose tissue. The findings showed that the tissue formed by the aggregate culture of the expanded ADSC population was less cartilaginous and that BMPCs may be a better choice for progenitor cell-based strategies for cartilage repair.

In the present study, three or four passages of BMSCs were induced for differentiation into osteogenic, adipogenic and neurogenic lineages *in vitro*, resulting in the formation of new bone and cartilage tissues *in vivo*. The deposition of calcium and an increased ALP activity were detected when the cells were incubated in an induction medium consisting of dexamethasone, ascorbic acid and β-GP. These conditions strongly suggest that BMSCs are able to differentiate into osteogenic cells. Round and translucent lipid vacuoles were detected in the cytoplasm and adipogenic differentiation was demonstrated when the cells were incubated in an induction medium containing indomethacin, dexamethasone, IBMX and human insulin. These molecules induced the MSCs to differentiate into adipocytes. IBMX promotes the adipocytic and neuroblastic differentiation of stem cells, whereas indo-methacin inhibits the neurogenic differentiation of MSCs. The MSCs were induced into neurogenic differentiation when basic fibroblast growth factor (bFGF), BME and IBMX were added to the basic medium. These findings demonstrated that BMSCs are able to differentiate into neuroblasts. The BMSCs were incubated in an osteogenic and a chondrogenic differentiation medium, seeded on a coral scaffold and implanted in mice *in vivo*. New bone and cartilage tissue formation was demonstrated *in vivo*.

In summary, in the present study, the BMSCs were incubated in osteogenic, adipogenic and neurogenic media to differentiate these cells *in vitro* into osteoblasts, adipocytes and neuroblasts, respectively, as well as to form new bone and cartilage tissues *in vivo*. The results showed that the fibroblast-like clone separated from the bone marrow of the ilium possesses the characteristics of stem cells. The study also demonstrated that the cells isolated from the bone marrow were homogeneous and that they were able to differentiate with high fidelity into osteogenic, adipogenic, neurogenic or chondrogenic lineages. These human BMSCs are also able to form bone and cartilage tissues when experimentally implanted *in vivo* and may thus be used as seed cells in bone tissue engineering.
